# Pulsed laser in liquid grafting of gold nanoparticle–carbon support composites

**DOI:** 10.3762/bjnano.16.26

**Published:** 2025-03-07

**Authors:** Madeleine K Wilsey, Teona Taseska, Qishen Lyu, Connor P Cox, Astrid M Müller

**Affiliations:** 1 Material Science Program, University of Rochester, Rochester, New York 14627, United States; 2 Department of Chemical Engineering, University of Rochester, Rochester, New York 14627, United States; 3 Department of Chemistry, University of Rochester, Rochester, New York 14627, United States

**Keywords:** catalysis, composite, electroreduction, gold nanoparticles, impedance

## Abstract

We developed a novel pulsed laser-assisted process for the fabrication of advanced composites of nonequilibrium gold nanoparticles on carbon fiber paper supports. Our one-step process integrates the generation of nanoparticles with their surface attachment and solves longstanding nanoparticle adhesion and electrical contact issues. Irradiation of hydrophilic carbon fiber paper submerged in aqueous HAuCl_4_ solution by nanosecond laser pulses produced composites with uniform distribution of gold nanoparticles on carbon fibers, taking advantage of the high internal surface area of carbon fiber paper. The pulsed laser-grafted composites exhibited zero measurable charge transfer resistance between gold nanoparticles and the carbon support, leading to superior cathode performance over conventionally prepared electrodes for electrocatalytic hydrogen evolution in aqueous bicarbonate reduction.

## Introduction

**Figure 1:**
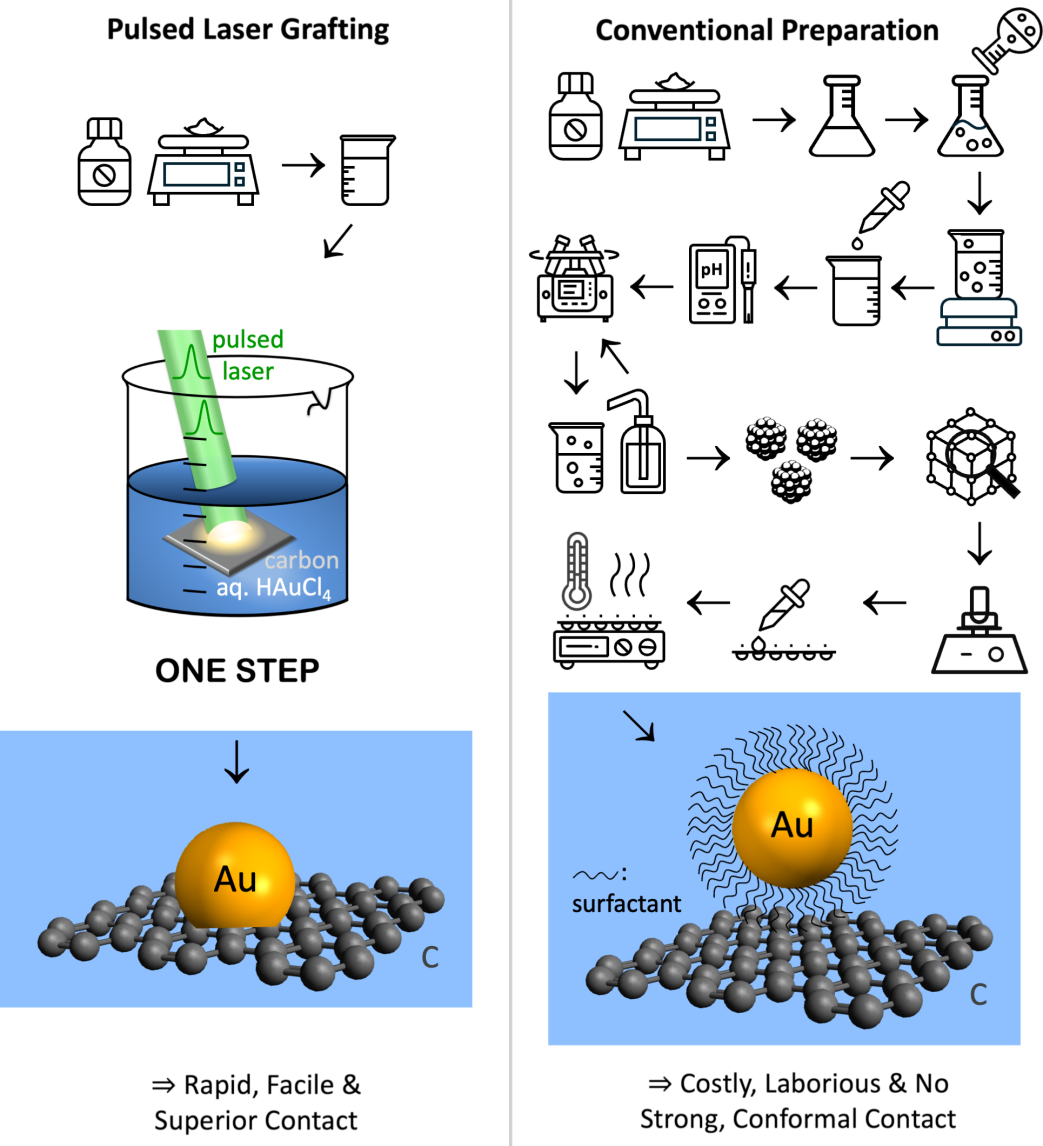
Schematic of pulsed laser grafting vs conventional preparation of gold nanoparticle–carbon support composites. Photographs of the actual pulsed laser grafting setup are shown in Supporting Information File 1, Figure S1. The illustration of the laser beam in Figure 1 was adapted from [1] (© 2021 R. C. Forsythe et al., published by ACS, distributed under the terms of the Creative Commons Attribution 4.0 International License, https://creativecommons.org/licenses/by/4.0).

The main challenge in the manufacturing of nanocatalyst-containing electrodes is the attachment of nanoparticles on electrode supports. Nanoparticles must be integrated with macroscopic supports to function as electrodes. A major obstacle in contemporary manufacturing of nanoparticle–support composites is their laborious inefficient multistep preparation, involving chemical synthesis, heating, cooling, collection, purification, distribution, and attachment on a support (Figure 1). Another challenge is the resulting poor physical and electrical contact of nanoparticles on supports. Our pulsed laser grafting process overcomes these problems by directly seeding and growing nanoparticles on substrates using nanosecond laser pulses, thereby eliminating the need for synthesizing, collecting, and attaching nanoparticles separately. This way, composite fabrication becomes more time-saving, cost-effective, and environmentally friendly.

A premier electrode support material is carbon because it is affordable, scalable, and stable under many electrochemical conditions [2]. On the laboratory scale, attachment is achieved electrostatically or by adding ion-conducting polymer (i.e., ionomer) binders to the nanoparticles, either as mixtures (inks) or overlayers [3–10]. Electrostatic attachment of nanoparticles to supports lacks long-term stability. A widely used ionomer binder is Nafion, which is highly acidic [11] and can corrode earth-abundant catalysts that are not acid-stable [12]. Ionomer binders can additionally lead to undesired side reactions, thus reducing the energy efficiency for the desired transformation [13]. Alkaline electrolytes can decrease acid-based side reactions but alter Nafion [11,14], impeding ion conductivity and overall performance [5]. Adding binders additionally complicates reaction mechanisms and introduces competing pathways or by-products [5]. Further challenges in traditional nanoparticle synthesis–attachment are long preparation times, the generation of hazardous organic solvent and ligand waste [15], and poor electrical contact at the nanoparticle–support interface, particularly for nanoparticles with surfactant-terminated surfaces [16]. Conventionally made nanoparticles rely on surfactants for size control [17–19]. Nanoparticles prepared by pulsed laser in liquid synthesis are surfactant-free [1], but the same binder strategies are used for nanoparticle–support composites as for conventionally made nanoparticles. Capping ligands and binders hinder intimate contact between nanoparticles and supports, lowering electrical contact fidelity and energy efficiency of the composite electrodes. Surfactants alter nanoparticle surfaces, complicating understanding and often lowering catalytic performance by blocking active sites. Surfactants (like binders) partake in electrochemical reactions and can create unwanted side products [7]. Long-term surfactant stability and associated catalyst agglomeration or detachment are another issue. Post-synthetic attachment of catalyst nanoparticles is poorly scalable, creates large amounts of organic hazardous waste, and results in wastage of unattached catalyst material, which is especially problematic with precious catalysts. Overall, separate nanoparticle synthesis–attachment produces composites with adhesion, durability, electrical contact, and concomitant energy efficiency issues.

Here, we report a new one-step pulsed laser grafting process that integrates the generation of nonequilibrium gold nanoparticles with their surface attachment on carbon fiber paper. This pulsed laser grafting approach solves longstanding adhesion and electrical contact issues. Instead of attaching ligand-capped nanoparticles to supports, nanoparticles are seeded and grown directly on the support using nanosecond laser pulses. In addition to enhancing adhesion and electrical contact in nanoparticle–carbon support composites, pulsed laser grafting enables the production of nonequilibrium nanoparticles. Laser-made nonequilibrium nanoparticles are kinetically trapped materials that are not accessible under thermodynamic equilibrium conditions [1]. Pulsed laser grafting permits access to extreme regions of materials phase diagrams by concentrating the laser energy into the region where nanoparticles emerge, followed by rapid cooling. Kinetically trapped nonequilibrium nanoparticles cannot be made by traditional syntheses [1]. Condensed matter systems, when driven far from equilibrium (by the laser pulse), can exhibit far more structural phases than their equilibrium counterparts [20].

We applied the pulsed laser-grafted nanoparticle–carbon fiber paper composites as cathodes in electrocatalytic aqueous bicarbonate reduction and compared their performance and electrical impedance to analogous electrodes that were conventionally prepared by electrostatically attaching commercial nucleation grown and citrate-capped gold nanoparticles to carbon fiber paper.

## Results and Discussion

**Figure 2:**
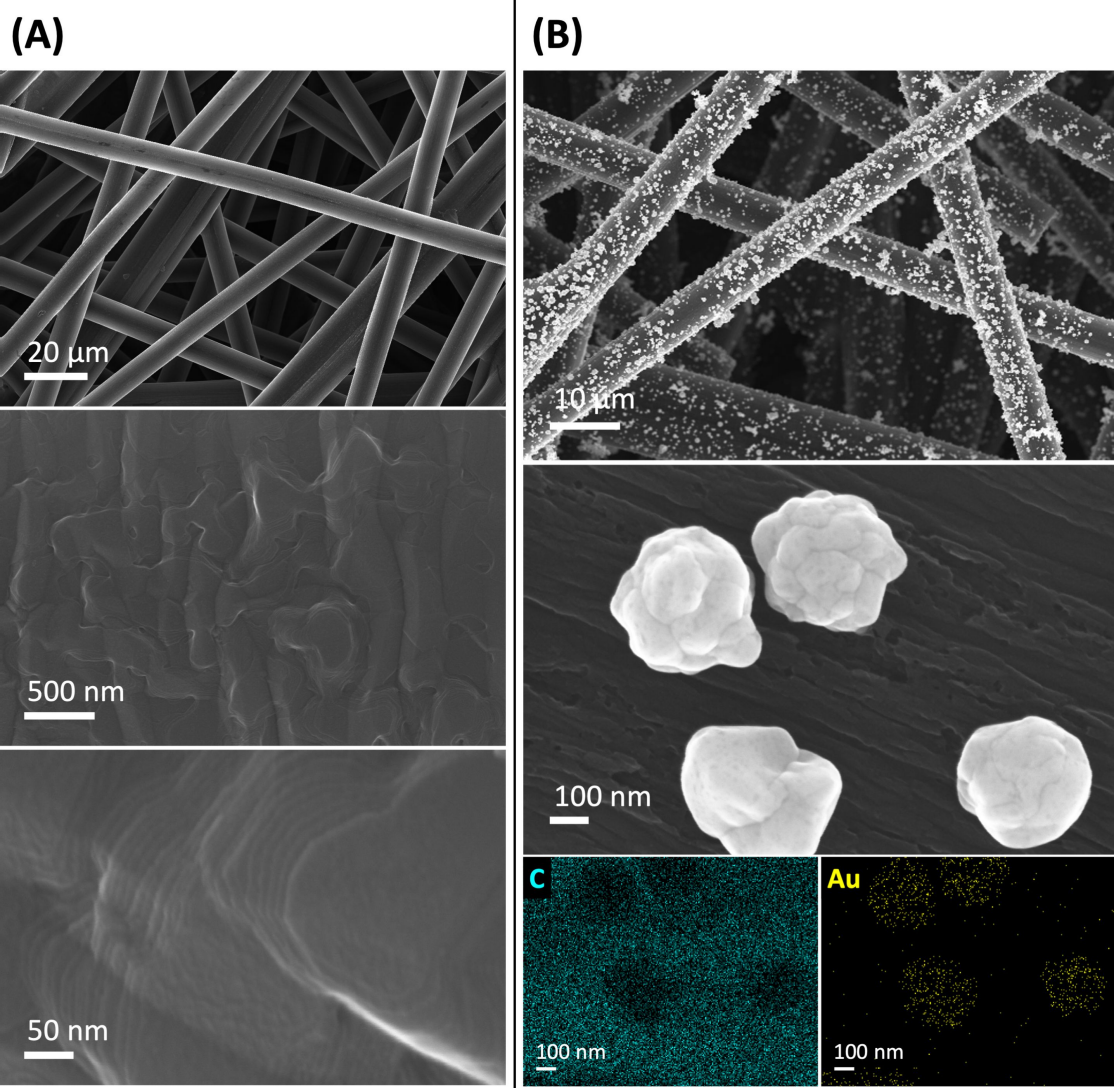
SEM images of hydrophilic carbon fiber paper (A) and pulsed laser-grafted gold nanoparticle–carbon fiber paper composites (B) with EDX maps showing carbon and gold.

Carbon fiber paper served as electrode support material because graphite is cost-effective, scalable, and the premier electrode support material for reduction reactions [21]. Gold nanoparticles were laser grafted from aqueous HAuCl_4_ solution. The use of an aqueous liquid requires that the carbon support is wettable by water. Making macroscopic carbon surfaces hydrophilic necessitates carbon surface oxygenates that are thermodynamically stable only at graphitic edges spaced closely enough to retain adsorbed water [22]. This precludes glassy carbon and basal-plane highly ordered pyrolytic graphite (HOPG); edge-plane HOPG is expensive, brittle, and not amenable to large electrode areas. In general, graphitic basal-plane carbon atoms are unreactive, unlike those in graphene, because of the π-stacking interactions of adjacent graphite sheets. We reported an environmentally friendly, rapid, scalable, acid-free process to make carbon fiber paper hydrophilic without destroying the carbon network, as other carbon fiber paper oxidation methods do [22], evident from scanning electron microscopy (SEM) imaging (Figure 2A). Hydrophilicity was achieved by graphitic edge carbon oxygenation and creating a high density of graphitic edges on the surfaces of carbon fibers [22]. Our process provides a carbon support material with retained porosity that does not slow mass transport in electrode processes [22], with a high surface area of carbon of 468 cm^2^ per geometric cm^2^ [23], derived from the reported specific surface area of the carbon fiber paper used here [24].

Pulsed laser grafting produced integrated gold nanoparticle–carbon fiber paper composites (Figure 2B), using aqueous 1.0 M HAuCl_4_ solution, hydrophilic carbon fiber paper, and unfocused Nd:YAG laser irradiation with 10 Hz, 8 ns, 532 nm, and 87 mJ·cm^−2^ pulses. We employed 532 nm pulses because gold nanoparticle generation works well at that wavelength, as nanoparticle nucleation and growth take advantage of this laser wavelength being resonant with the surface plasmon resonance in gold nanoparticles [25]. For 532 nm nanosecond pulses, graphite has an effective absorption coefficient of 5 µm^−1^ [26], resulting in an ablation threshold fluence of 0.7 J·cm^−2^ [27]; thus, our chosen fluence was well below this ablation threshold. The critical melting fluence of graphite has been reported to be 0.13 J·cm^−2^ [28], suggesting that our laser fluence did not enable carbon sublimation. Stable gold colloids have been produced by reactive nanosecond laser irradiation of aqueous [AuCl_4_]^–^ solutions [29–30]. Colloidal gold nanoparticle formation occurred by nucleation of reduced (metallic) gold atoms [25,31–32]. As in pulsed laser in liquid synthesis [1], the nanoparticles resulting from reactive pulsed laser processing are surfactant-free.

**Figure 3:**
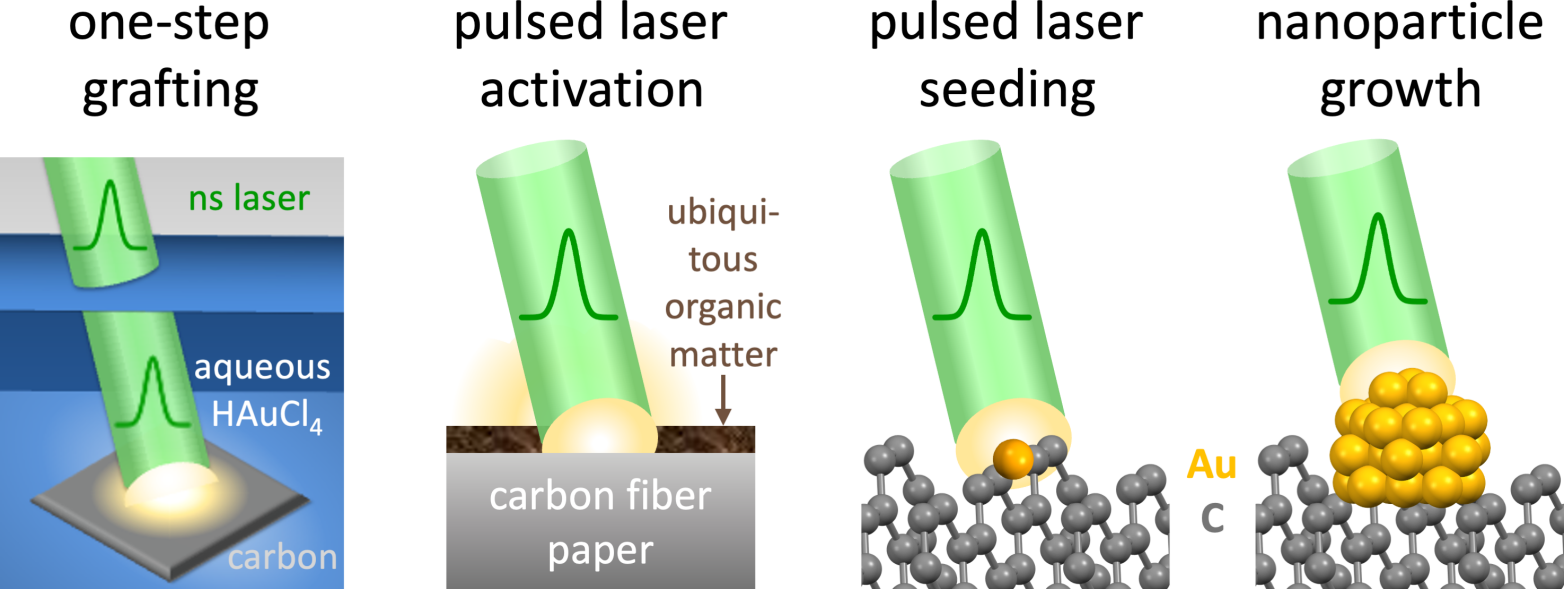
Schematic of the processes during pulsed laser in liquid grafting. The illustration of the laser beam in Figure 3 was adapted from [1] (© 2021 R. C. Forsythe et al., published by ACS, distributed under the terms of the Creative Commons Attribution 4.0 International License, https://creativecommons.org/licenses/by/4.0).

We used nanosecond laser pulses to minimize surface damage to the graphitic carbon fiber paper. Based on the thermal time constants of graphite, a few nanoseconds are required to dissipate heat over lengths of the order of micrometers [33]. This makes nanosecond pulses ideal to limit nonlinear excitation effects. By taking advantage of the well-understood processes of reactive pulsed laser processing for colloid formation [25,31–32], we successfully grafted gold nanoparticles directly on submerged hydrophilic carbon fiber paper supports. The laser grafting process took 60 min, significantly less time than typical chemical gold nanoparticle synthesis and subsequent support attachment of several hours [34]. The use of nanosecond pulses in the pulsed laser grafting process has the additional advantage of enabling pulsed laser decontamination and activation of surfaces, while concurrently exploiting the advantages of nanoparticle preparation by pulsed laser in liquid synthesis, including the many degrees of control to fabricate tailored nanomaterials [1]. Pulsed laser grafting advances the mature technology of pulsed laser cleaning [35–36] by simultaneously activating the support surface, to seed and grow nanoparticles immediately on the surface that is briefly trapped in this decontaminated/activated state (illustrated in the schematic in Figure 3). Organic deposits are ubiquitous at materials surfaces. These organics impede the adhesion and electrical contact of nanoparticles, even when the nanoparticles are surfactant-free. Regular nanosecond pulsed laser cleaning rids surfaces of organic deposits by the interaction of nanosecond laser pulses with surface contaminants via absorption and volatilization [35–36]. However, surfaces return to their initial state fast and cannot be kept microscopically clean because the removal of ubiquitous surface contaminants leaves unterminated, highly reactive surfaces with dangling bonds and, as a result, high surface energy, which is lowered by surface reconstruction [37]. Passivation of dangling bonds on graphite occurs fast [38]; we were unable to find a reported timescale. In general, dangling bonds possess short lifetimes. For example, dangling OH bonds of water have sub-picosecond lifetimes [39], whereas the lifetimes of dangling bonds in solid materials are of the order of nanoseconds to microseconds [40]. For comparison, typical timescales expected for laser-induced growth of Au nuclei are hundreds of femtoseconds to a few nanoseconds [41], and the metal–cluster nucleation rate for the formation of ca. 5 nm gold nanoparticles was reported as 3.0 × 10^−6^ s^−1^ [25]. This suggests that pristine graphite surfaces, generated by the in situ decontamination and activation by nanosecond laser cleaning, can exist for Au embedding during the pulsed laser grafting process. However, this microscopic surface decontamination and activation is too short-lived to prevail on the timescales of conventional nanoparticle–support composite preparation of seconds to hours. Therefore, conventionally prepared composite materials generally suffer from microscopic surface contaminations that impair the physical and electrical contact of nanoparticles on supports. We surmise that our in situ decontaminated/activated carbon surfaces enabled immediate embedding of small nucleation sites, at which the laser made nanoparticles grow, resulting in superior physical and electrical contact at the nanoparticle–support interface (shown below). We used nanosecond laser pulses for more efficient and less damaging surface decontamination compared to shorter pulses [42]. Besides, picosecond or femtosecond pulses are more expensive, creating obstacles for future scaled up composite manufacturing applications.

The gold nanoparticles were uniformly distributed on the carbon fibers and penetrated into the three-dimensional network of the carbon fibers (Figure 2B), thus taking full advantage of the high internal surface area of carbon fiber paper. The gold nanoparticles were grafted at internal carbon fiber paper surfaces, as expected with laser light scattering by the carbon fibers. The pulsed laser-grafted gold nanoparticles exhibited cauliflower morphology with approximately 200 nm diameter and no detectable carbonaceous shells (Figure 2B and cf. X-ray photoelectron spectroscopy (XPS) data below). Assembly of gold nanoparticles by nanosecond laser pulses in liquids has been reported [43]. A generation of similar concave edges occurs in twinning. Twinned gold nanoparticles have been found to exhibit enhanced electrocatalytic activity in reductions because of an increased number of undercoordinated surface sites [44].

**Figure 4:**
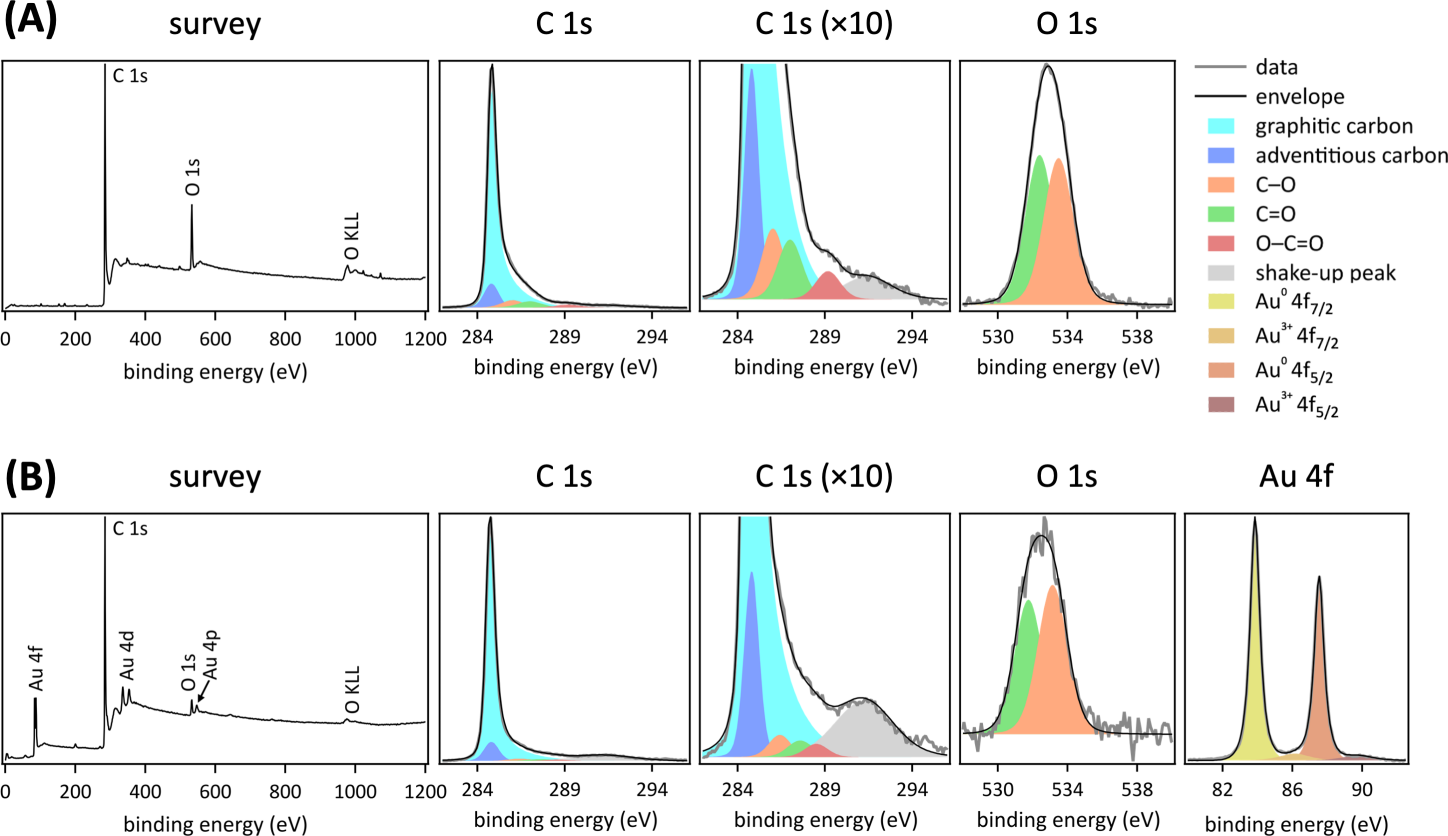
XPS data of hydrophilic carbon fiber paper (A) and pulsed laser-grafted gold nanoparticle–carbon fiber paper composite (B). Relative contents of XPS species are given in Supporting Information File 1, Table S1.

XPS data corroborate the absence of a carbonaceous shell on the gold nanoparticles, evident from the detection of surface gold (Figure 4). The elements carbon and oxygen are present in hydrophilic carbon fiber paper (Figure 4A), as expected for this support material [22]. High-resolution C 1s region spectra required six peaks to fit the data, including an asymmetric peak and a shake-up peak, in keeping with previously reported XPS data of graphitic carbon [45–48]. The asymmetric peak with a central binding energy range of 284.5 to 285.0 eV was assigned to graphitic carbon, in agreement with reported values [49–50]. We additionally observed adventitious carbon, with a central binding energy of 284.8 eV [51]. That left three peaks, assignable to oxygenates, with C 1s central binding energy values of (286.5 ± 0.5), (287.5 ± 0.5), and (288.5 ± 0.5) eV, attributed to C–O, C=O, and O–C=O, respectively [52–58]. Two peaks were needed to fit the high-resolution O 1s region spectra with central binding energies of (532.0 ± 0.5) eV, consistent with C=O functional groups, and (533.5 ± 0.5) eV, assigned to C–O species [52–55], in keeping with prior data for hydrophilic carbon fiber paper [22–23]. Element-specific relative sensitivity factors resulting from photoemission cross sections and analyzer transmission of photoelectrons were used to determine atom percentages [22]. The measured O 1s atom percentages for each component were used to constrain the respective C 1s peak fits. The characteristic C 1s π–π* shake-up peak was larger and broadened in the pulsed laser-grafted gold nanoparticle–carbon fiber paper composite, compared to hydrophilic carbon fiber paper (Figure 4), indicating disorder and contributions from sp^2^- and sp^3^-hybridized carbon [58], as expected from the nanosecond laser treatment that enabled the embedding of gold atoms.

Gold was additionally present in XPS data of the pulsed laser-grafted gold nanoparticle–carbon fiber paper composites (Figure 4B). High-resolution Au 4f data were fitted using a Gaussian–Lorentzian doublet with an orbital splitting of (3.5 ± 0.14) eV of Au 4f_7/2_ and Au 4f_5/2_, using the same full width half maximum within the range of 0.5–1.5 eV and a peak area ratio constrained to 4:3, in agreement with reported values [59]. Four component peaks were required to fit the measured data. The predominant species was metallic gold at a binding energy of (84.4 ± 0.1) eV for Au 4f_7/2_ and (88.0 ± 0.1) eV for Au 4f_5/2_ [7]. Additionally, peaks corresponding to the Au^3+^ oxidation state were observed at (87.0 ± 0.2) eV and (90.5 ± 0.2) eV corresponding to Au 4f_7/2_ and Au 4f_5/2_ of Au_2_O_3_, a result of oxide formation at the nanoparticle surface upon exposure to air [60–61]. Attempts to include an Au^+^ component with a central binding energy of 85.2 eV [62] did not match the measured data, excluding the presence of Au^+^ here. The presence of Au^+^ has been observed in 800 nm femtosecond-reactive laser ablation in aqueous HAuCl_4_ solution [41]. We did not find evidence for Au–C bonds in the XPS data, likely because the generated gold nanoparticles obscured any Au–C bonds that may have formed at the gold–carbon interface. XPS is surface-sensitive, with a typical probe depth limited to the top few nanometers of a material [63]. Therefore, underground (under the gold nanoparticles) spectral signatures were not observable here.

**Figure 5:**
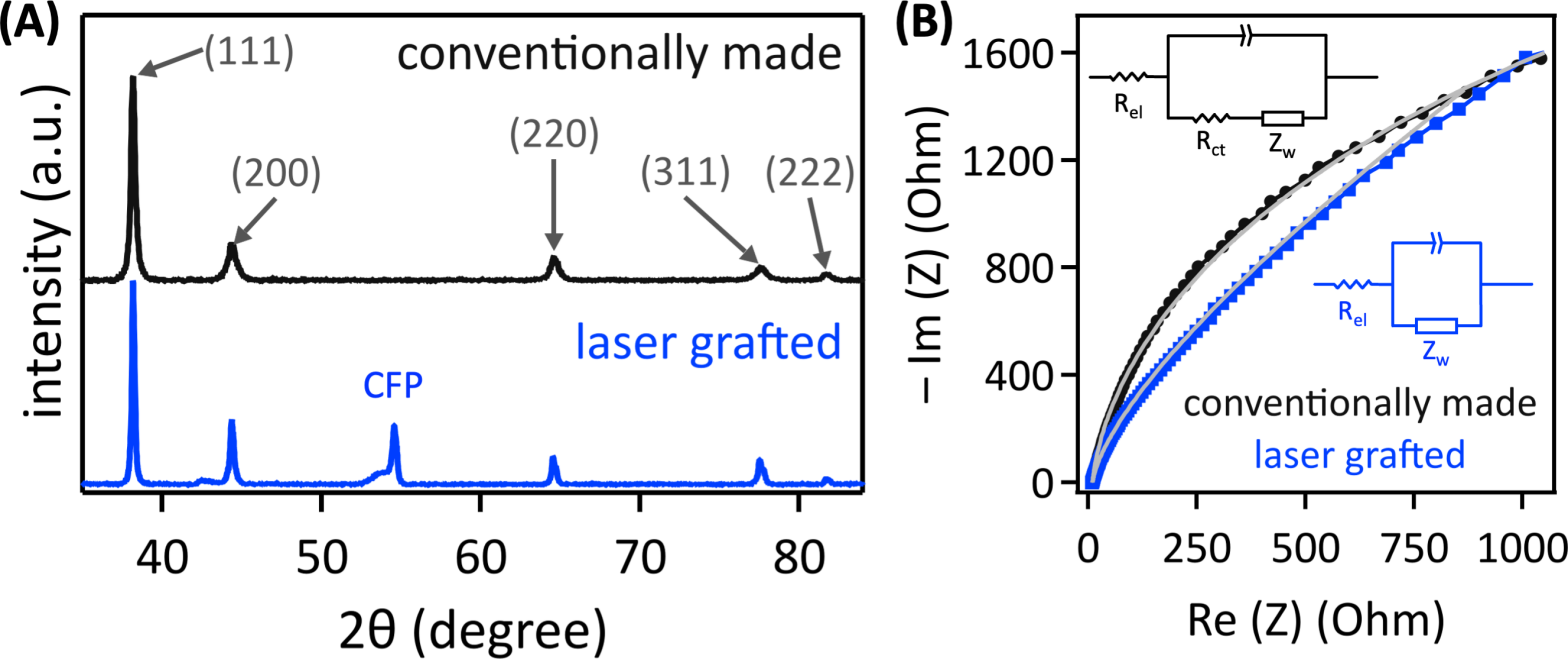
XRD data of gold nanoparticles (A). EIS data, with gray fits using the inset equivalent circuit models of gold nanoparticle–carbon fiber paper composites (B).

Pulsed laser grafting created nonequilibrium gold nanoparticle–carbon fiber paper composites, evident from powder X-ray diffraction (XRD) data (Figure 5A). We normalized the XRD patterns to the (111) peak and found that the (200) or (311) peak maxima of laser-grafted gold nanoparticles were 1.8 or 1.7 times higher than that of analogous conventionally prepared gold nanoparticles, respectively, indicating nonequilibrium faceting, consistent with the observed cauliflower morphology. The (220) and (222) peak heights did not change as a function of gold nanoparticle preparation method.

The pulsed laser-grafted integrated composites showed superior electrical contact compared to analogous electrodes with chemically synthesized gold nanoparticles with citrate surfactants that were electrostatically attached to hydrophilic carbon fiber paper, evident from electrochemical impedance spectroscopy (EIS) data (Figure 5B). Impedance, measured in an electrochemical setup, is the time-dependent opposition to alternating current stemming from the combined effect of ohmic resistance, capacitance, and phase elements in an electronic circuit. Impedance is a quantitative measure for electrical contact between nanoparticles and supports [64].

EIS data, visualized in a Nyquist plot, graph the negative imaginary impedance (*Z*) vs the real impedance [2]. Modeling the data with an electronic circuit that reflects the electrochemical system gives values for resistances (*R*) and capacitances at all interfaces and the electrolyte. In our EIS measurements, the most relevant circuit element is the charge transfer resistance (*R*_ct_) between the gold nanoparticles and the graphitic carbon support, measured at open circuit potential so that electrochemical reactions do not obfuscate the electrical characteristics [2]. The other circuit elements are the electrolyte resistance (*R*_el_, the *x*-axis intercept), which was small because of the high ionic conductivity of the 1.0 M aqueous KHCO_3_ electrolyte, the Warburg impedance (*Z*_w_) due to mass transport limitations of the redox species to the electrode, and the ubiquitous capacitance at the interface between the electrode and the electrolyte. This capacitance is non-ideal at the porous, non-flat gold nanoparticle–carbon fiber paper electrode, necessitating incorporation of a constant phase element. In a Nyquist plot, semi-circles indicate the presence of resistance and capacitance in parallel. A larger semi-circle radius indicates more resistance and capacitance, and ergo inferior electrical contact. EIS data that consist of diagonal lines show capacitances only and negligible *R*_ct_.

The EIS data of the pulsed laser-grafted gold nanoparticle–carbon fiber paper composites showed zero measurable charge transfer resistance (Figure 5B) and, therefore, excellent electrical contact. In contrast, chemically synthesized gold nanoparticles with citrate surfactants, electrostatically attached to hydrophilic carbon fiber paper with high surface area, showed inferior electrical contact, with a charge transfer resistance of 2.87 kΩ (Figure 5B), which is consistent with published values for conventionally prepared gold–carbon composites [65].

**Figure 6:**
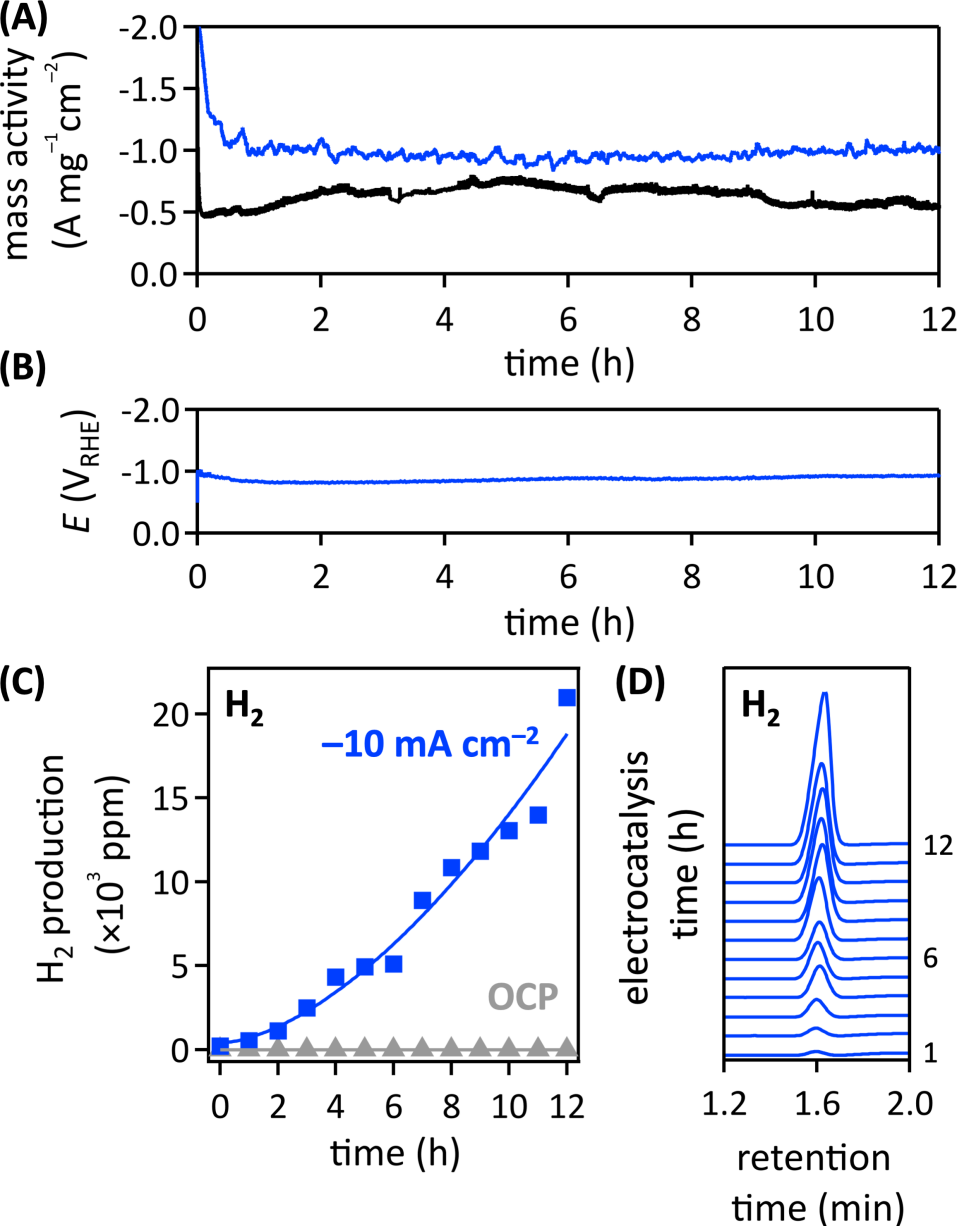
Electrocatalytic aqueous bicarbonate reduction data from pulsed laser-grafted (blue) or conventionally prepared (black) gold nanoparticle–hydrophilic carbon fiber paper composites. Mass activity, expressed as current normalized to the mass of gold, determined from ICP-MS data, and geometric area of the cathode, at a constant applied potential of −1.3 V vs RHE (A). Potential needed to maintain a constant current density of −10 mA·cm^−2^ (B). Electrocatalytic product generation; blue: at a constant current density of −10 mA·cm^−2^, gray: at open circuit potential (C). GC data as a function of electrocatalysis time (D). The lines are fits (blue, power law; gray, linear). The full GC traces and additional GC data are shown in Supporting Information File 1, Figures S2–S4.

We expected that our finding of zero measurable charge transfer resistance of the pulsed laser-grafted gold nanoparticle–carbon fiber paper composite would lead to enhanced electrocatalytic performance. Therefore, we applied the laser-grafted gold nanoparticle–carbon fiber paper composites as cathodes for electrocatalytic bicarbonate reduction in aqueous 0.5 M KHCO_3_, pH 8.3, electrolyte (Figure 6). Gas chromatography (GC) was used to identify products. In mildly alkaline electrolytes, as used here, gold-catalyzed aqueous bicarbonate reduction to hydrogen has been reported [66–67].

Our electrocatalysis testing revealed increased durability and electrochemical performance of pulsed laser-grafted over conventionally prepared cathodes. Chronoamperometry data, collected at a constant applied potential of −1.3 V vs RHE, showed enhanced stability and mass activity of the pulsed laser-grafted gold nanoparticle–carbon fiber paper composite, compared to an analogous conventionally prepared cathode (Figure 6A). We attribute the superior performance of pulsed laser-grafted electrodes to the improved adhesion and electrical contact and concomitant absence of charge transfer resistance between the grafted gold nanoparticles and the carbon fiber paper support. Generated currents were normalized to the geometric area of the cathode and the mass of gold, which we obtained from digesting the electrodes in aqua regia and collecting inductively coupled plasma mass spectrometry data. We note that only gold was quantified, as carbon has a *Z* number that is too low for ICP-MS detection [68]. Pulsed laser-grafted and conventionally prepared electrodes had a mass loading of 70 and 8.5 µg·cm^−2^_geometric_, respectively. The diameters of laser-grafted and commercial gold nanoparticles were 200 and 100 nm, respectively. The larger size of laser-grafted gold nanoparticles is not an issue for the evaluation of the bicarbonate mass activity because larger gold nanoparticles have been found to be inferior reduction catalysts [69], especially gold nanoparticles larger than 10 nm [70]. Hence, we err on the side of underestimating the benefits of pulsed laser grafting for gold nanoparticle–carbon fiber paper composite fabrication. The mass activity of pulsed laser-grafted cathodes was a factor of 1.65 higher than that of conventionally prepared electrodes (Figure 6A).

Chronopotentiometry data, collected in an H-cell at a constant current density of −10 mA·cm^−2^, corroborated the exceptional stability of the pulsed laser-grafted gold nanoparticle–carbon fiber paper composites (Figure 6B). Further, we did not observe any gold loss with respect to carbon in EDX data before and after 2 h of electrocatalysis (Supporting Information File 1, Figure S5). GC data show that pulsed laser-grafted cathodes produced predominantly hydrogen in aqueous bicarbonate reduction. Minor amounts of CO_2_ were additionally detected in the GC data, likely from the reaction of protons with bicarbonate. No other gases were detected (cf. Supporting Information File 1, Figures S2–S4). GC data of a control experiment at open circuit potential, where no faradaic current flows [2], did not show product generation. This demonstrates that the gold nanoparticle–carbon fiber paper composite cathode electrocatalyzed hydrogen evolution in aqueous bicarbonate reduction (Figure 6C,D). Hydrogen can arise from the reduction of water (2H_2_O + 2e^−^ ⇌ H_2_ + 2OH^−^) or bicarbonate (2HCO_3_^−^ + 2e^−^ ⇌ H_2_ + 2CO_3_^2−^) in aqueous 0.5 M KHCO_3_, pH 8.3, electrolyte [66], where water reduction is kinetically more sluggish [71]. When protons are present, CO_2_ can be formed from bicarbonate in a non-faradaic reaction via HCO_3_^−^ + H^+^ ⇌ CO_2_ + H_2_O. Protons are generated by water oxidation at the anode (2H_2_O ⇌ O_2_ + 4H^+^ + 4e^−^) [12]. To enable equilibration of bicarbonate anions between the two compartments during electrolysis, we used an anion exchange membrane to separate the cathode from the anode compartment. Although anion exchange membranes are designed to primarily allow the passage of anions, proton transport or leakage, can occur because of the inherent structure and presence of water within the membrane [72–74]. This way, anodically generated protons can cross over into the cathode compartment and produce CO_2_ from bicarbonate. At a constant current density of −10 mA·cm^−2^, at which proton-generating water oxidation occurred at the anode, we detected a small amount of CO_2_ in the GC data of the cathode headspace (Supporting Information File 1, Figure S2 and Figure S4). We observed an exponential asymptotic growth of CO_2_ that reached steady state after 3 h (Supporting Information File 1, Figure S4), likely due to buffering of protons in the aqueous bicarbonate electrolyte. Mixtures of H_2_ and CO_2_ are valuable precursors in carbon dioxide hydrogenation to produce synthetic fuels and chemicals [75–76].

Overall, our novel one-step aqueous pulsed laser grafting process enables the fabrication of surfactant-free nonequilibrium gold nanoparticles directly on carbon fiber paper supports, solving longstanding adhesion and electrical contact issues of metal nanoparticle–support composites. This new nanosecond laser-based composite manufacturing methodology is more rapid and efficient than existing processes because it obviates the heating, cooling, and separation steps of traditional chemical nanoparticle syntheses. It additionally eliminates post-synthetic attachment of catalyst nanoparticles that results in wastage of unattached catalyst material, which is especially problematic with precious catalysts, and it does not require binders. The pulsed laser grafting process is predicated on in situ nanosecond pulsed laser decontamination and activation of the support surface to create short-lived pristine graphite surfaces, at which gold is seeded and grows into grafted nanoparticles by reactive pulsed laser in liquid synthesis. Because of this dual mechanistic role of the nanosecond laser, we expect that our new composite fabrication methodology can be expanded beyond gold nanoparticles by taking advantage of the vast chemical flexibility of reactive pulsed laser in liquid nanomaterial fabrication [1]. Reductive and oxidative solution chemistries are widely available and well investigated in wet chemistry contexts [17,77–81]. Our novel ability to utilize general solution chemistry toolkits to prepare tailored nanoparticles, integrated with laser-induced seeding on pulsed laser decontaminated/activated support surfaces and followed by laser-enabled nanoparticle growth, provides universality and simplicity. As a result, pulsed laser grafting has broad applications in sustainable manufacturing, decarbonization technologies, catalysis, sensing, and biomedical fields.

## Conclusion

Pulsed laser grafting of gold nanoparticle–carbon fiber paper composites presents a significant advancement in electrode design for electrocatalytic applications. Our novel one-step aqueous pulsed laser grafting process enables the fabrication of surfactant-free gold nanoparticles directly on carbon fiber paper supports by integrating nanoparticle synthesis and attachment, obviating typical laborious nanoparticle synthesis, separation, and immobilization procedures. Pulsed laser-grafted composites exhibited zero measurable charge transfer resistance between gold nanoparticles and the carbon support. In addition to the exceptional electrical contact, these composites have enhanced durability and electrochemical performance, surpassing conventionally prepared electrodes, showcasing a 1.65 times higher mass activity and exceptional stability in aqueous bicarbonate reduction to hydrogen. The efficiency of pulsed laser grafting of nanoparticles on supports and its applicability across various fields underscore its potential for sustainable manufacturing of electrodes for catalysis.

## Experimental

All chemicals were used as received. Deionized water with a resistivity of ≥17.5 MΩ·cm was obtained from a Thermo Scientific Barnstead Smart2Pure Pro UV/UF 15 LPH Water Purification System. The experiments were performed at room temperature and in ambient air. Glassware was cleaned with aqua regia, thoroughly rinsed with water, and dried before use. Data analysis and graphing were conducted using Igor Pro 8.04 (Wavemetrics), unless otherwise stated.

### Composite preparation

Hydrophilic carbon fiber paper was prepared as support for gold nanoparticles. Details of the process to render carbon fiber paper hydrophilic are described elsewhere [22]. In short, as purchased carbon fiber paper (FuelCell Store, AvCarb MGL190) was sonicated in 1.0 M aqueous sodium dodecyl sulfate solution (AG Scientific, ≥99%) and subsequently electrooxidized at +1.63 V vs Ag/AgCl for 20 min in aqueous 0.1 M KHCO_3_ solution (pH 8.3, Alfa Aesar, 99.7–100.5%).

Laser-grafted gold nanoparticle–carbon fiber paper composites were prepared using pulsed laser in liquid synthesis. Hydrophilic carbon fiber paper was placed on a glass flange in a 30 mL beaker and submerged in 12 mL of an aqueous 1.0 M solution of gold(III) chloride trihydrate (Sigma Aldrich, ≥99.9%), 5 mm below the liquid surface. The solution was stirred at 750 rpm. Dissolved reactant concentrations in the range from micromoles to a few moles have been used in pulsed laser in liquids synthesis [1]. An unfocused 532 nm, 8 ns pulsed laser beam from a 10 Hz Nd:YAG laser (Spectra-Physics Quanta-Ray LAB-190) with a pulse energy of 680 mW was directed into the beaker. After 60 min, the laser-grafted composite was removed and rinsed with water.

Conventional gold nanoparticle–carbon fiber paper composites were prepared by immersing a 2.4 cm (wide) × 3.8 cm (long) piece of hydrophilic carbon fiber paper, placed on the bottom of a custom-made Teflon tub, in 2.0 mL of commercially available aqueous colloid of citrate-capped gold nanoparticles (100 nm, nanoComposix), followed by drying under a heat lamp at 60 °C for 20 min.

### Physical characterization

Scanning electron microscopy (SEM) images were obtained at UR-Nano. A Zeiss Auriga scanning electron microscope with a Schottky field-emission emitter was operated at 20.00 kV with a working distance of 4.9 mm. Energy-dispersive X-ray (EDX) spectroscopy data were collected using an SEM-integrated EDAX Octane elect plus spectrometer with a with silicon drift detector. Double sided carbon tape was used to adhere the gold nanoparticle–carbon fiber paper composites to sample stubs.

X-ray photoelectron spectra (XPS) data were collected at UR-Nano using a Kratos Axis Ultra XPS instrument with a monochromatized Al Kα source. At a base pressure of 3.0 × 10^−8^ mbar, the instrument operated at 200 W and 15 kV. Samples were washed with water, dried, and affixed to double-sided adhesive copper tape. Survey scans were averaged over five scans and spanned 0–1200 eV with a 1 eV step size, 200 ms dwell time, and 160 eV pass energy. High-resolution core level scans were averaged over five scans and measured with a 0.1 eV step size, 260 ms dwell time, and 20 eV pass energy. All spectra were referenced against the adventitious C 1s peak at 284.8 eV [51]. The data processing, including Shirley background subtraction and Gaussian/Lorentzian peak fitting, was performed in CasaXPS (Version 2.3.24) using instrument-specific atomic sensitivity factors.

X-ray diffraction (XRD) measurements were conducted at the Chemical Analysis Lab at the Rochester Institute of Technology using a Bruker D8 ADVANCE diffractometer with Cu Kα radiation (40 kV and 40 mA). The configuration included a 0.6 mm primary slit, a 5.0 mm secondary slit, and a 2.5 mm anti-scatter screen, and a Lynxeye detector. Each measurement was performed with a resolution of 0.020° in 2θ and 0.5 s per step dwell time, resulting in approximately 40 min per sample. Background subtraction was performed using Bruker DIFFRAC.SUITE software.

Inductively coupled plasma mass spectrometry measurements were conducted at the University of Rochester Medical Center. A Perkin Elmer NexION 2000 system featuring multielement detection and parts per billion/parts per trillion sensitivity was used. The gold nanoparticle–carbon fiber paper composites were digested in aqua regia, prepared from concentrated Aristar Plus trace metal grade nitric acid (VWR, 69% w/w) and hydrochloric acid (VWR, 37% w/w).

### Electrochemistry

Electrical impedance spectroscopy (EIS) data were collected at open circuit potential in aqueous 1.0 M KHCO_3_, pH 8.3, electrolyte. A standard one-compartment three-electrode setup was used. Laser-grafted or conventionally made gold nanoparticle–carbon fiber paper composites with a geometric area of 0.49 cm^2^ served as working electrodes, platinum mesh as counter electrode, and a reversible hydrogen electrode (Gaskatel Hydroflex) as reference electrode. A Bio-Logic potentiostat (8-slot VSP3e potentiostat/galvanostat/EIS system) was used. The sinusoidal perturbation for EIS was set to an amplitude of 10 mV, with a frequency range spanning from 100 kHz to 100 mHz. The resolution was set to 40 points per decade with each point being an average of five measurements. The EIS spectra were analyzed using the Bio-Logic EC-Lab software package.

Electrocatalytic bicarbonate reduction data were acquired in aqueous 0.5 M KHCO_3_, pH 8.3, electrolyte. The working electrodes were pulsed laser-grafted or conventionally prepared gold nanoparticle–hydrophilic carbon fiber paper composites with 0.49 cm^2^ geometric area. Conventionally prepared composites were fabricated by immobilizing commercial gold nanoparticles (100 nm, nanoComposix, 50 µg·mL^−1^ in 2 mM aqueous sodium citrate) on hydrophilic carbon fiber paper, with a gold loading of 10 µg·cm^−2^_geometric_. All electrochemical data were acquired without iR compensation. Chronoamperometry data were collected for 12 h at −1.3 V vs RHE, provided by a Bio-Logic 8-slot VSP3e potentiostat, in a standard single-compartment three-electrode cell, with the electrolyte stirred at 500 rpm. The counter electrode was platinum mesh, and the reference electrode was a reversible hydrogen electrode (Gaskatel HydroFlex^®^). Chronopotentiometry data were collected for 12 h at −10 mA·cm^−2^, provided by a Bio-Logic SP-150-EIS potentiostat, in a custom-made small-gap H-cell obtained from the Jaramillo group at Stanford University [82]. A control experiment was performed at open circuit potential. The two compartments of the H-cell with 9 mL electrolyte each were separated by a Selemion anion exchange membrane (AMV-N). Pulsed laser-grafted gold nanoparticle–hydrophilic carbon fiber paper composite served as working electrode. The counter electrode was a Pt foil (Aldrich, 0.025 mm thick, 99.9%), and an Ag/AgCl (BaSi) reference electrode, calibrated against a reversible hydrogen electrode (Gaskatel HydroFlex^®^), was used. Produced gas was detected by an in-line gas chromatograph (GC, SRI, Multi-Gas #5 configuration) connected to the 2 mL headspace of the working electrode compartment of the electrochemical cell. Hydrogen was detected by a thermal conductivity detector, and a flame ionization detector equipped with a methanizer was used to detect all other gases. Following a published procedure [82], the gas chromatograph was programmed to collect a chromatogram every 20 min. A certified standard calibration gas (Airgas) was used to calibrate the gas chromatograph.

## Supporting Information

File 1 Photographs of the pulsed laser grafting setup, GC data, EDX spectra, and relative contents of XPS species.

## Data Availability

All data that supports the findings of this study is available in the published article and/or the supporting information of this article.
