# Supplementary material for: Pulsed laser in liquid grafting of gold nanoparticle–carbon support composites
Source: Beilstein J Nanotechnol. 2025 Mar 7;16:349–61. doi: 10.3762/bjnano.16.26 (PMC11897648; doi:10.3762/bjnano.16.26)
Supplement: File 1 — Photographs of the pulsed laser grafting setup, GC data, EDX spectra, and relative contents of XPS species. [file Beilstein_J_Nanotechnol-16-349-s001.pdf]

BEILSTEIN JOURNAL OF NANOTECHNOLOGY

Supporting Information

for

Pulsed laser in liquid grafting of gold nanoparticle–carbon support composites

Madeleine K. Wilsey, Teona Taseska, Qishen Lyu, Connor P. Cox and Astrid M. Müller

Beilstein J. Nanotechnol. **2025,** *16,* 349–361. doi:10.3762/bjnano.16.26

Photographs of the pulsed laser grafting setup, GC data, EDX spectra, and relative contents of XPS species

License and Terms: This is a supporting information file under the terms of the Creative Commons Attribution License (https://creativecommons.org/licenses/by/4.0). Please note that the reuse, redistribution and reproduction in particular requires that the author(s) and source are credited and that individual graphics may be subject to special legal provisions.
The license is subject to the *Beilstein Journal of Nanotechnology* terms and conditions: (https://www.beilstein-journals.org/bjnano/terms)

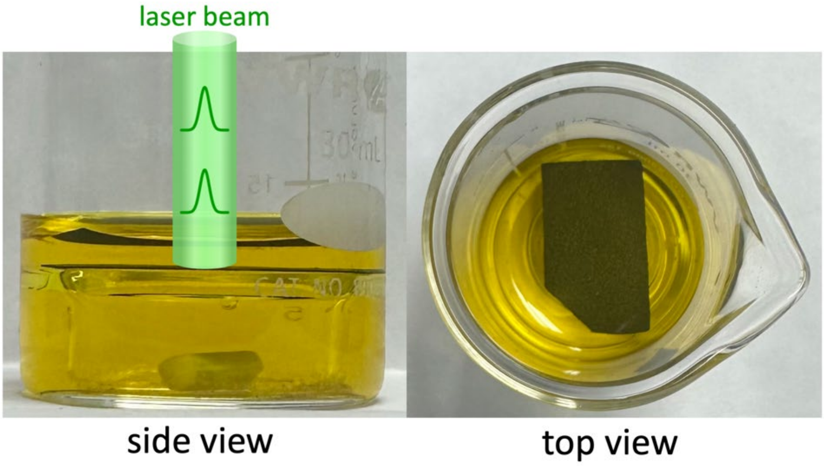

Figure S1: Photographs of the pulsed laser grafting setup for fabricating gold nanoparticles on hydrophilic carbon fiber paper from aqueous $HAuCl_4$ solution. In the side view, the total reflection of the carbon fiber paper is visible as a photography artifact.

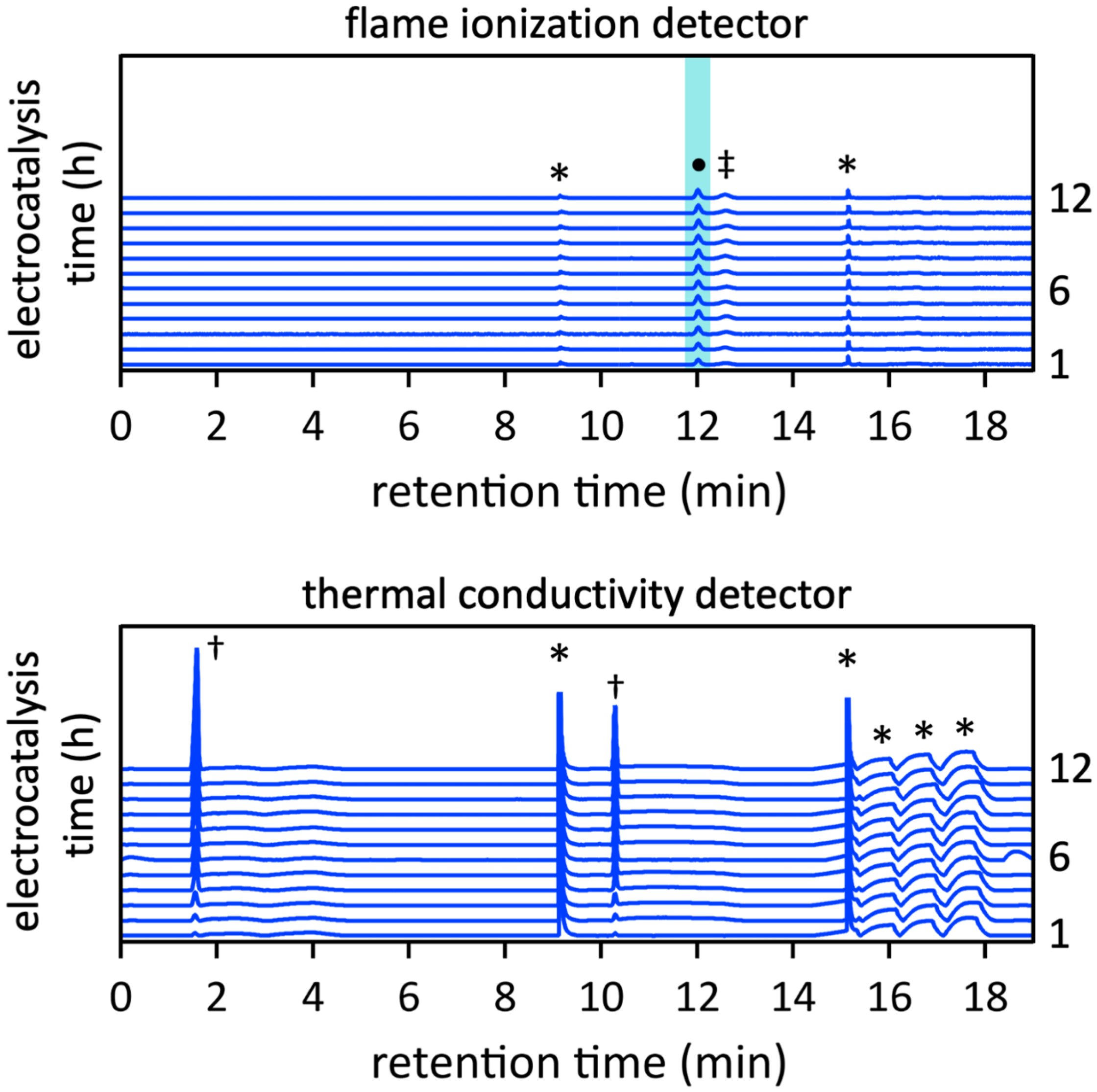

Figure S2: GC data as a function of electrocatalysis time for electrocatalytic aqueous bicarbonate reduction at −10 mA·cm^{-2}. Symbols: (•) denotes CO_2 detection (highlighted in cyan, peak areas are shown in Figure S3), (†) denotes H_2 detection, (*) denotes signals due to valve switches, and (‡) denotes GC artifacts.

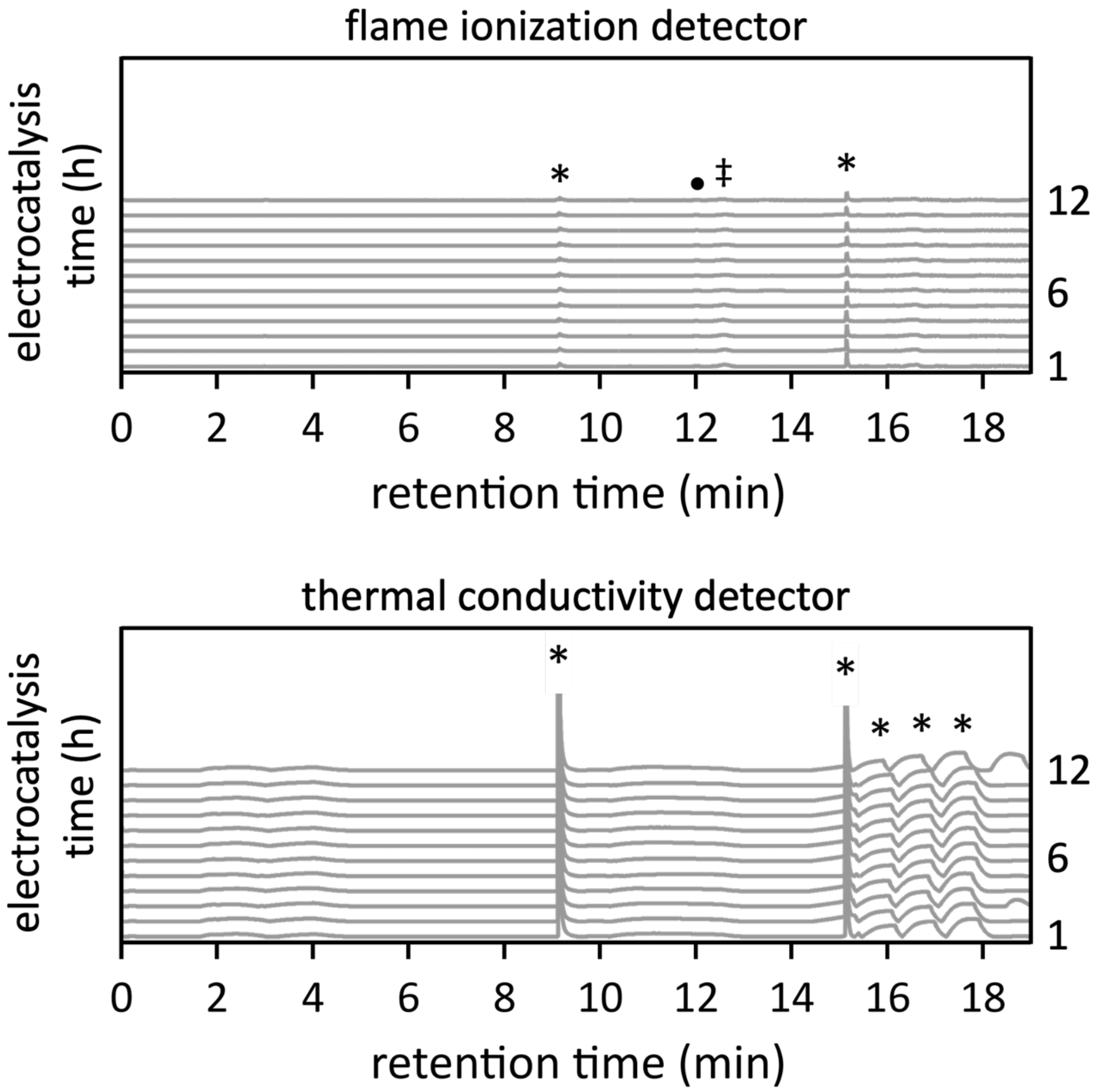

Figure S3: GC data as a function of electrocatalysis time for electrocatalytic aqueous bicarbonate reduction at open circuit potential. Symbols: (•) denotes CO_2 detection, (*) denotes signals due to valve switches, and (‡) denotes GC artifacts.

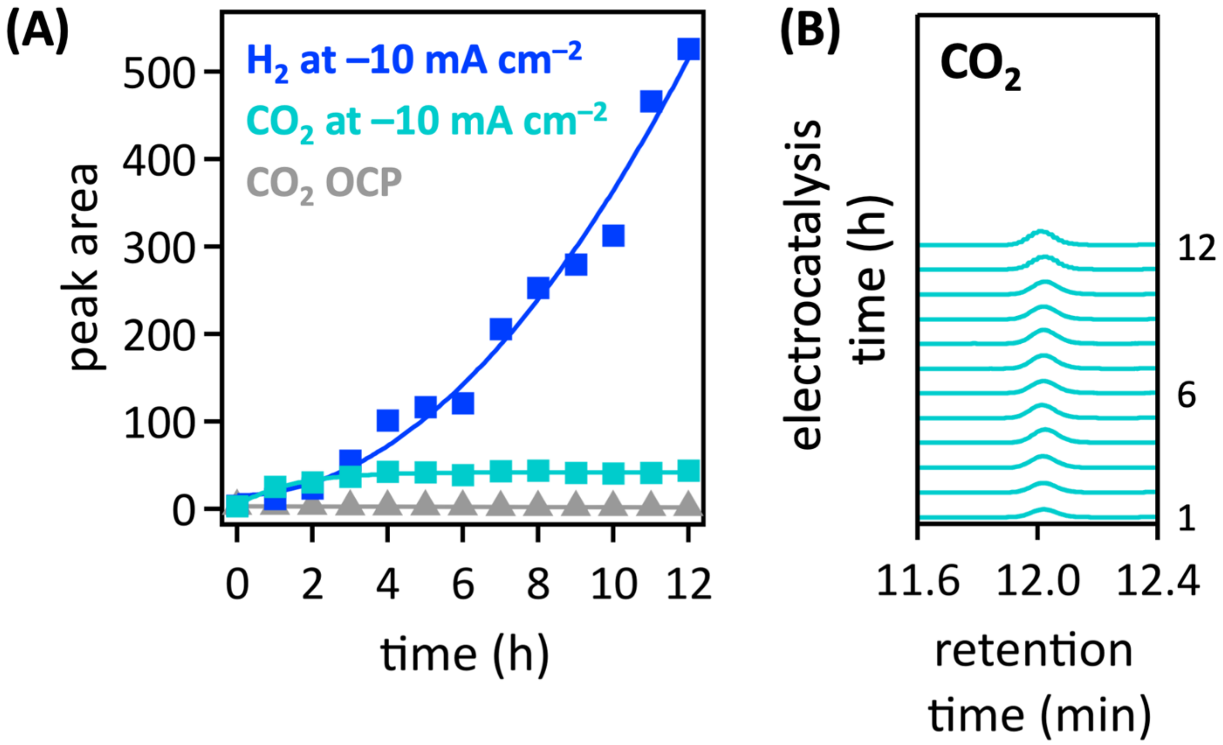

Figure S4: Electrocatalytic product generation; colors: blue, H_2 at a constant current density of −10 mA·cm^{-2}, cyan, CO_2 at a constant current density of −10 mA·cm^{-2}, gray, CO_2 at open circuit potential (OCP). Markers, data; lines, fits using the following functions: blue, power law, cyan, exponential asymptotic, gray, line (A). GC data for CO_2 at a constant current density of −10 mA·cm^{-2} as a function of electrocatalysis time (B).

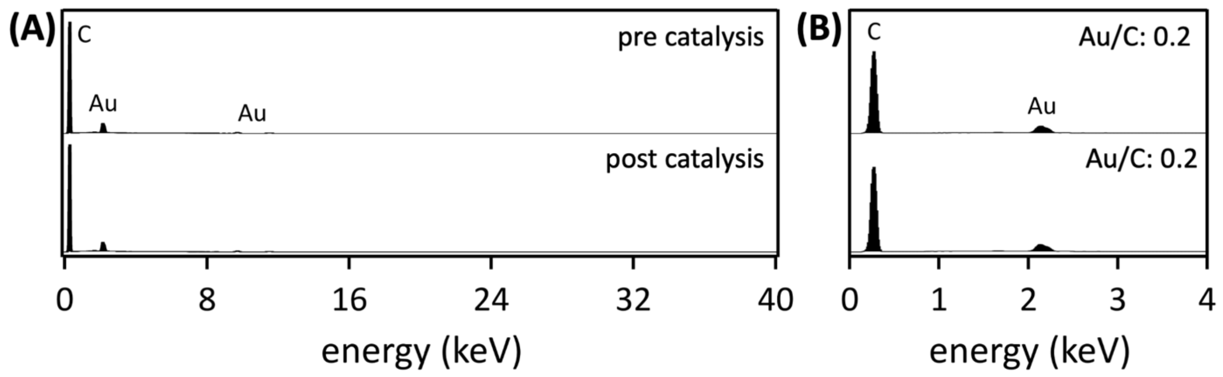

Figure S5: EDX spectra of pulsed laser grafted gold nanoparticle–hydrophilic carbon fiber paper composites before and after 2 h of electrocatalysis at a constant current density of −10 mA·cm^{-2}, showing the full energy axis (A) and an expanded energy axis (B).

Table S1: Relative contents of surface carbon oxygenates, the π–π* shake-up peak, and gold species with respect to the sum of graphitic and adventitious carbon content.

species	hydrophilic carbon fiber paper		laser grafted gold nanoparticle–hydrophilic carbon fiber paper composites	
	central binding energy (eV)	relative content (atom %)	central binding energy (eV)	relative content (atom %)
graphitic carbon	284.7	90.2	284.7	90.4
adventitious carbon	284.8	9.8	284.8	9.6
C=O	286.0	6.0	286.4	1.7
C–O	287.0	5.2	287.6	1.5
O–C=O	288.9	2.0	288.5	1.1
π–π* shake-up peak	291.1	4.8	291.2	10.5
Au^0	–	–	84.3	1.4
Au^{3+}	–	–	86.4	0.1
Au^0	–	–	88.0	1.1
Au^{3+}	–	–	90.3	0.1
